# Article Correction: We Are on the Verge of Breakthrough Cures for Type 1 Diabetes, but Who Are the 2 Million Americans Who Have It?

**DOI:** 10.36469/001c.134140

**Published:** 2025-04-18

**Authors:** Rebecca Smith, Samara Eisenberg, Aaron Turner-Phifer, Jacqueline LeGrand, Sarah Pincus, Yousra Omer, Fei Wang, Bruce Pyenson

**Affiliations:** 1 Milliman, Inc., New York, NY, USA; 2 Breakthrough T1D, Washington, DC, USA

**Keywords:** population health, diabetes forecast, continuous glucose monitor, insulin pump, administrative claims, type 1 diabetes, demographic projection, real-world data, socioeconomic determinants

## Abstract

**Background:**

Two million Americans have type 1 diabetes (T1DM). Innovative treatments have standardized insulin delivery and improved outcomes for patients, but patients’ access to such technologies depends on social determinants of health, including insurance coverage, proper diagnosis, and appropriate patient supports. Prior estimates of US prevalence, incidence, and patient characteristics have relied on data from select regions and younger ages and miss important determinants.

**Objectives:**

This study sought to use large, nationally representative healthcare claims data sets to holistically estimate the size of the current US population with T1DM and investigate geographic nuances in prevalence and incidence, patient demographics, insurance coverage, and device use. This work also aimed to project T1DM population growth over the next 10 years.

**Methods:**

We used administrative claims from 4 sources to identify prevalent and incident T1DM patients in the US, as well as various demographic and insurance characteristics of the patient population. We combined this data with information from national population growth projections and literature to construct an actuarial model to project growth of the T1DM population based on current trends and scenarios for 2024, 2029, and 2033.

**Results:**

We estimated 2.07 million T1DM patients nationally across all insurance coverages in our 2024 baseline model year: 1.79 million adults (≥20 years) and 0.28 million children. This represents a US T1DM prevalence rate of 617 per 100 000 and an incidence rate of 0.016%. By 2033, we project the US population with T1DM will grow by about 10%, reaching approximately 2.29 million patients.

**Discussion:**

Our results showed important differences in T1DM prevalence and incidence across regions, payers, and ethnic groups. This suggests studies based on more geographically concentrated data may miss important variation in prevalence and incidence across regions. It also indicates T1DM prevalence tends to vary by income, consistent with several international studies.

**Conclusions:**

Accurate projections of T1DM population growth are critical to ensure appropriate healthcare coverage and reimbursement for treatments. Our work supports future policy and research efforts with 2024, 2029, and 2033 projections of demographics and insurance coverage for people with T1DM.

## BACKGROUND

The 2022 publication of global statistics for type 1 diabetes mellitus (T1DM) highlights the need for better statistics on the 2 million Americans with the condition.^1^ Recent technological advancements that customize T1DM therapies on a minute-by-minute patient basis produce improved outcomes for people with T1DM, and research points to potential cures.^2^ Better care will need to overcome stereotypes, such as T1DM’s legacy as “juvenile diabetes,” confusion with the more common type 2 diabetes mellitus (T2DM), and incomplete demographic profiles based on limited registry information. Furthermore, US demographic and insurance coverage changes will affect population health efforts to reach people with T1DM. This paper fills gaps in US T1DM information with 2024, 2029, and 2033 projections of demographic and insurance coverage for people with T1DM.

The isolation of insulin in 1921 marked a transformative moment for what is now known as T1DM, turning it from a tragic and rapidly fatal childhood disease into a survivable chronic condition, although one with significant comorbidities and excess mortality.^3,4^ Recent progress has led to broad adoption of continuous glucose monitors (CGM), portable electronic devices that track and monitor glucose levels in real time, and wearable insulin pumps, which have reduced comorbidity burdens and increased survival rates for people with T1DM.^5^ Soon, we may have treatments to delay, prevent, and even reverse the autoimmune processes that cause T1DM.^2^

Diabetes refers to several illnesses involving the body’s metabolization of sugar via insulin, a pancreatic enzyme. This paper focuses on T1DM, where the pancreas stops making insulin.^6^ T1DM becomes fatal quickly without regular injections of insulin. Because T1DM was primarily diagnosed in children, it was originally called “juvenile diabetes.” Type 2 diabetes, which is more prevalent and often associated with obesity, occurs when the pancreas produces insufficient insulin or ineffectively uses insulin for sugar metabolism. In both forms of diabetes, fluctuations in blood sugar (poor glycemic control) are associated with comorbidities. Although T1DM and T2DM are very different diseases, many information sources do not distinguish between the conditions; patient diagnoses may be miscoded, and patients’ forms of diabetes may not be obvious from their treatments.^7,8^

About 2 million Americans have T1DM—much lower than the nearly 30 million with T2DM.^9^ Diabetes is the eighth-leading cause of death in the United States, with about 9% of deaths coming from T1DM.^10^ T1DM is predominantly diagnosed in children and young adults but similarly across sexes.^6^ Patients with T1DM have mortality rates 3 to 18 times higher than standard.^11^ Innovative treatments and technologies have helped standardize insulin delivery and improve outcomes for patients. A variety of automated devices are in use, such as CGMs and insulin pumps, but patients’ access to such devices depends on insurance coverage, proper diagnosis, and appropriate patient supports, all of which are affected by social determinants of health.^12^

Our use of real-world data, which includes some key socioeconomic drivers, provides important public health information about the 2 million Americans with T1DM. Historically, estimates of T1DM prevalence and incidence have come from epidemiological, clinic-based, population-based, prospective birth, case cohort, and cross-sectional studies.^13^ By contrast, we used several large, nationwide payer-based administrative data sets combined with estimates of incidence changes and US demographic projections by region and ethnicity to produce 5-and 10-year forecasts. We found that the number of T1DM patients is higher than other estimates, with important regional and socioeconomic differences, some of which are expected to widen over time. Finally, we replicated others’ findings of significant T1DM incidence among older adults, including those covered by Medicare.^14^

This study aimed to holistically estimate the size of the current US population with T1DM and investigate geographic nuances in prevalence and incidence, patient demographics, insurance coverage, and device use and to project T1DM population growth over the next ten years.

We hope forecasting the demographic details of America’s population with T1DM will help public health, payer, and advocates’ efforts to spread best practices and, optimistically, future cures.

## METHODS

This study utilized real-world administrative claims data (which insurers and others collect when they process bills from healthcare providers during their payments for covered services, devices, and drugs,) to create estimates of the US T1DM population in 2019, segmented by type of insurance coverage (eg, commercial, Medicaid, Medicare, uninsured). We used these segmented estimates to project T1DM prevalence across the entire US population. The data also informed mortality and incidence rates for the T1DM population, which was combined with US population forecasts to produce a 10-year population projection. All data was processed and analyzed using SAS version 9.4.

### Claims Analysis

We analyzed 2018 to 2020 data from several large administrative databases (**Appendix Exhibit A1**).^15–17^ These databases consisted of the Medicare 100% Research Identifiable File (approximately 21 million lives for Fee-for-Service and approximately 21 million lives for Medicare Advantage),^18^ Merative MarketScan (approximately 11 million lives for commercial plans),^19^ and the Milliman Health Sources Database (approximately 4 million lives for Medicaid).^20^ The data used for commercial insurance includes claims and enrollment from approximately 100 payers, and the data used for Medicaid contains longitudinal claims and enrollment from several national and regional health plans across all states. Estimates of T1DM patients for commercial populations were assessed using MarketScan, Managed Medicaid (MCD) using Milliman Consolidated Health Cost Guidelines^TM^ Sources Database (CHSD), and Medicare Fee-for-Service (FFS) and Medicare Advantage (MA) using the Research Identifiable File (RIF) data. Patients were required to have at least 1 month of enrollment between January 2018 and December 2020, except for the MA population, for which data were available only through December 2019. The commercial and Medicaid populations were restricted to those under 65 years old. For purposes of incidence calculation, patients were required to have 6 months of exposure before their first T1DM diagnosis.

Insulin-using patients were identified using 3 or more distinct claims for insulin, insulin pumps, or insulin-related supplies on separate dates, at least 30 to 120 days apart. This identification used Healthcare Common Procedure Coding System (HCPCS) and National Drug Code (NDC) codes (**Appendix Exhibit A2-A3**). Patients with fewer than 12 months of continuous enrollment following their first date of insulin use were excluded from the analysis.

Patients with T1DM were then identified from among the insulin-using population based on evidence of at least 1 *International Classification of Diseases, Tenth Revision, Clinical Modification* (ICD-10-CM) diagnosis code for T1DM (**Appendix Exhibit A4**) in any diagnosis code position and at least 1 claim for insulin, an insulin pump, or a related supply (**Appendix Exhibit A2-A3**) within 90 days before or after the patient’s first claim with a T1DM diagnosis during the study period. Patients’ index dates were set to the date of their first claim with a T1DM diagnosis. Patients demonstrating evidence of drugs or drug combinations specific to type 2 diabetes mellitus (T2DM) (**Appendix Exhibit A5**) at any time during the study period were excluded. This exclusion considered sulfonylureas, thiazolidinediones, glucagon-like peptide-1 agonists (GLP-1s), sodium-glucose cotransportor-2 (SGLT2s), dipeptidyl peptidase 4 (DPP-4s), meglitinides (glinides), and α-glucosidase inhibitors (AGIs) given the years analyzed.^8^ Additionally, patients with evidence of only long-acting insulin prescriptions (**Appendix Exhibits A2 and A3**) were excluded, as T1DM patients generally require short-or intermediate-acting insulins; however, patients using premixed insulin formulations including a short-or intermediate-acting insulin (**Appendix Exhibit A2 and A3**) were included.

Patients were flagged as newly diagnosed with T1DM (incident) if there was no evidence of a T1DM diagnosis (**Appendix Exhibit A4**) or use of insulin, insulin-related durable medical equipment, or an insulin pump (**Appendix Exhibit A2 and A3**) in the 6 months leading up to their index date. This analysis was possible only among patients with at least 6 months of continuous medical and pharmacy coverage before their T1DM index date. This identification approach is similar to other published and validated algorithms.^8,21^

Patient counts were compiled for each data set based on insurance coverage (commercial, MCD, FFS, MA), age, sex, geographic region, and metropolitan statistical area (MSA) status. For the Medicare populations, additional variables were captured, including race/ethnicity and dual-Medicare-Medicaid eligibility status, the latter indicating low-income beneficiaries. Patients’ claims were also examined for the presence of at least 1 HCPCS or NDC code indicating use of CGMs and/or insulin pumps during the study period, such that utilization rates for these devices could be summarized.

Patients were grouped into 5-year age bands, with wider bands for the youngest and oldest ages. We used 4 geographic regions (**Appendix Exhibit A6**) and split residence into MSA (urban) or not (rural). Sex was captured from enrollment data, and race/ethnicity categories tabulated included African American, Hispanic, non-Hispanic White, and all other races (available only for Medicare).

### Extrapolation of Claims Data to National Estimates

Prevalence rates were determined for each of the coverages analyzed and extrapolated to national counts for 2019 (**Appendix Exhibits A1 and A7**). The claims-derived prevalence rates were applied to population or enrollment totals obtained from CMS, the Kaiser Family Foundation, the Centers for Disease Control and Prevention (CDC), the US Department of Veterans Affairs, and the CMS Chronic Conditions Data Warehouse. All sources reported enrollment for 2019.^22–27^ In addition to the 4 coverage types directly examined via claims, we developed prevalence rates for Veterans Affairs (VA), Uninsured, and Other Medicare (individuals with either only Medicare Part A or only Part B). The commercial prevalence rate was used for the VA population, the Managed MCD prevalence rate for all MCD populations and Uninsured, and the FFS prevalence rate for the Other Medicare population.

For modeling, claims-derived patient counts were used to calculate agent weights for each combination of demographic factors within each insurance coverage. The agent weight for any combination of demographic factors was determined by the proportion of T1DM patients in a given coverage, considering factors such as sex, age, region, race, dual status, and MSA status, in relation to the entire nationalized T1DM population. These weights played a crucial role in allocating the national patient counts into coverage-specific cohorts based on age, sex, demographic, and geographic factors. For example, one cohort is the population of commercially insured African American males aged 30 to 34 years living in the South in an MSA area. As with prevalence figures, we used the commercial coverage weights as proxies for the VA population, Medicaid weights as proxies for the Uninsured population, and FFS for the Other Medicare population.

T1DM incidence rates were computed across coverage-specific age, sex, demographic, and geographic cohorts. For rates of T1DM device use, patients in each cohort were categorized based on their usage of CGMs, insulin pumps, both CGMs and pumps, or neither device. T1DM age distributions, incidence rates by age, and device use rates by age were smoothed by fitting curves to initially developed rates derived from the data.

### 10-Year Projections

Events within a given year, such as device uptake or death, were modeled in a probabilistic manner. We developed mortality loads for the T1DM population relative to standard population mortality separately for ages 0 to 64 years and for 65 years and over using FFS data. Only FFS data were used due to lack of death date information in commercial claims and because FFS data was available for a longer time horizon than MA data. We examined raw counts, categorized by age and sex, of patients alive in 2020 who subsequently died in 2021 within the eligible denominator population (those with Medicare Parts A, B, and D in 2020) and within the T1DM population. Expected death counts were then calculated for the same population under the assumption that mortality would follow the 2019 standard mortality tables from CDC WONDER.^28^ Initial mortality loads for the T1DM population and the standard population were determined by calculating the ratios of the total raw death counts in each respective population to the number of deaths assuming standard mortality. Finally, the impact of COVID-19 was removed by calculating the ratio of the initial T1DM mortality load to the standard mortality load (**Appendix Exhibits A8-A9**).

We note that our method of using Medicare-based mortality loads for commercial or Medicaid T1DM patients may overstate expected deaths because under-65 Medicare T1DM patients may suffer from disabling conditions such as end-stage renal disease.

The number of new cases of T1DM in the model were calibrated to produce a baseline, steady state model by age, which considered mortality and age progression. The baseline model maintained consistency in demographics for the T1DM population over the 10-year projection. Population growth was then incorporated into that model using US population growth projections^29^ (**Appendix Exhibits A10-A11**) and T1DM incidence growth from the Global Burden of Disease Study 2017.^30^

Mortality for patients newly starting on CGMs or insulin pump devices reflected the mortality-reducing impacts of these technologies.^31–34^ For patients using both devices, excess T1DM mortality over standard mortality was reduced by 50%, meaning that the T1DM mortality rate was reduced by 50% of the T1DM mortality rate in excess of the standard mortality rate for a given age and sex (**Appendix Exhibit A12**). This improvement was applied in the first year of new device use and all subsequent years. For patients exclusively using CGMs, a 40% reduction was applied. For those exclusively using pumps, a 10% mortality reduction was applied. The model also assumed 85% of the population would use devices by Model Year 3, compared with approximately 78% in 2019.

## RESULTS

### 2024 Baseline

We estimated 2.07 million T1DM patients nationally across all insurance coverages in our 2024 baseline model year –1.79 million adults (≥20 years) and 0.28 million children (**Table 1**). This represents a US T1DM prevalence rate of 617 per 100,000 (**Figure 1**) with an average age of 47. The majority of patients (68%) were classified as non-Hispanic White, and the largest proportion were covered by commercial (47%), followed by Medicare (FFS, MA, and Other Medicare populations totaling 29%) and Medicaid (15%) insurance coverage (**Table 2**). We observed 78% of patients with CGMs and/or insulin pump devices.

**Table 1.** Model Years 1, 5, and 10: Type 1 Diabetes Projections by Demographic Group and Device Use

**T1DM Patient Population Group**	**Model Year**			**Cumulative % Change**		
	**1**	**5**	**10**	**Years 1-5**	**Years 5-10**	**Years 1-10**
Total population (N)	2 072 557	2 148 483	2 289 197	3.7	6.5	10.5
By payer (N)						
Commercial	973 020	983 365	1 008 562	1.1	2.6	3.7
Medicaid	311 021	345 386	404 094	11.0	17.0	29.9
Medicare Fee-for-Service	362 655	356 818	359 743	-1.6	0.8	-0.8
Medicare Advantage	210 443	230 923	262 413	9.7	13.6	24.7
Veterans Affairs	23 883	24 423	26 494	2.3	8.5	10.9
Uninsured	157 063	171 856	189 568	9.4	10.3	20.7
Medicare Part A only or Part B only	34 472	35 713	38 322	3.6	7.3	11.2
By sex (N)						
Female	1 031 497	1 075 807	1 152 395	4.3	7.1	11.7
Male	1 041 060	1 072 676	1 136 802	3.0	6.0	9.2
By age band, y (N)						
<10	56 280	60 787	77 708	8.0	27.8	38.1
10-14	96 241	99 259	108 754	3.1	9.6	13.0
15-19	129 387	131 059	135 831	1.3	3.6	5.0
20-24	140 183	140 724	142 202	0.4	1.1	1.4
25-29	144 392	144 962	146 677	0.4	1.2	1.6
30-34	148 645	149 347	151 345	0.5	1.3	1.8
35-39	152 902	153 776	156 162	0.6	1.6	2.1
40-44	157 155	158 236	161 089	0.7	1.8	2.5
45-49	161 402	162 811	166 477	0.9	2.3	3.1
50-54	165 348	167 217	171 791	1.1	2.7	3.9
55-59	162 579	164 825	170 394	1.4	3.4	4.8
60-64	159 402	163 020	171 854	2.3	5.4	7.8
65+	398 640	452 461	528 914	13.5	16.9	32.7
By region (N)						
Midwest	529 932	548 840	581 709	3.6	6.0	9.8
Northeast	400 337	410 041	432 965	2.4	5.6	8.2
South	749 807	772 540	814 895	3.0	5.5	8.7
West	392 481	417 063	459 627	6.3	10.2	17.1
By race (N)						
African-American	327 663	344 542	370 007	5.2	7.4	12.9
Hispanic	268 984	286 829	317 191	6.6	10.6	17.9
Non-Hispanic White	1 403 788	1 438 405	1 513 058	2.5	5.2	7.8
All other race	72 122	78 707	88 940	9.1	13.0	23.3
By dual status (n)						
Dual	160 448	170 590	187 326	6.3	9.8	16.8
Non-dual	447 122	452 864	473 152	1.3	4.5	5.8
By MSA status						
MSA	1 880 100	1 954 650	2 091 173	4.0	7.0	11.2
Non-MSA	192 457	193 833	198 024	0.7	2.2	2.9
Average age (y)	47.2	48.1	48.9	1.8	1.7	3.5

**Figure 1.**
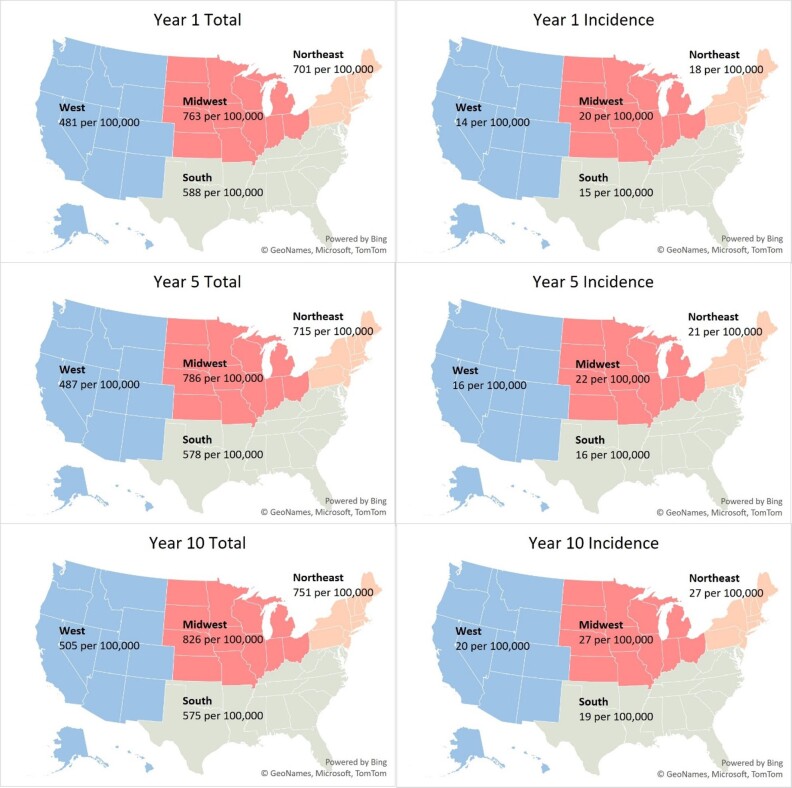
Variation of Type 1 Diabetes Incidence and Prevalence by Geography in Model Years 1, 5, and 10.

**Table 2.** US Model Year 1 Type 1 Diabetes Patient Count Projections by Age, Sex, and Insurance Coverage

**Sex**	**Age Band**	**Total**	**COM**	**MCD**	**FFS**	**MA**	**VA**	**UNI**	**Other MCR**
Male, total	All ages	1 041 060	520 423	150 344	170 579	94 792	12 774	75 920	16 228
Male	<10	29 460 (2.8%)	14 777 (2.8%)	9529 (6.3%)	0 (0.0%)	0 (0.0%)	365 (2.9%)	4789 (6.3%)	0 (0.0%)
Male	10-14	48 713 (4.7%)	26 639 (5.1%)	14 239 (9.5%)	0 (0.0%)	0 (0.0%)	655 (5.1%)	7180 (9.5%)	0 (0.0%)
Male	15-19	69 069 (6.6%)	40 238 (7.7%)	18 501 (12.3%)	0 (0.0%)	0 (0.0%)	988 (7.7%)	9342 (12.3%)	0 (0.0%)
Male	20-24	72 562 (7.0%)	50 412 (9.7%)	13 685 (9.1%)	284 (0.2%)	2 (0.0%)	1237 (9.7%)	6915 (9.1%)	27 (0.2%)
Male	25-29	71 822 (6.9%)	47 610 (9.1%)	14 484 (9.6%)	1075 (0.6%)	64 (0.1%)	1168 (9.1%)	7319 (9.6%)	102 (0.6%)
Male	30-34	75 361 (7.2%)	47 265 (9.1%)	15 356 (10.2%)	3095 (1.8%)	435 (0.5%)	1160 (9.1%)	7759 (10.2%)	292 (1.8%)
Male	35-39	78 344 (7.5%)	48 249 (9.3%)	14 230 (9.5%)	5691 (3.3%)	1263 (1.3%)	1184 (9.3%)	7190 (9.5%)	537 (3.3%)
Male	40-44	82 206 (7.9%)	49 947 (9.6%)	13 392 (8.9%)	7759 (4.5%)	2383 (2.5%)	1226 (9.6%)	6767 (8.9%)	732 (4.5%)
Male	45-49	85 192 (8.2%)	51 497 (9.9%)	11 736 (7.8%)	9913 (5.8%)	3916 (4.1%)	1264 (9.9%)	5930 (7.8%)	935 (5.8%)
Male	50-54	86 850 (8.3%)	51 399 (9.9%)	10 118 (6.7%)	11 874 (7.0%)	5965 (6.3%)	1261 (9.9%)	5112 (6.7%)	1120 (6.9%)
Male	55-59	83 780 (8.0%)	47 310 (9.1%)	8763 (5.8%)	12 948 (7.6%)	7951 (8.4%)	1161 (9.1%)	4428 (5.8%)	1 219 (7.5%)
Male	60-64	80 550 (7.7%)	45 079 (8.7%)	6312 (4.2%)	13 648 (8.0%)	9930 (10.5%)	1106 (8.7%)	3188 (4.2%)	1 287 (7.9%)
Male	65+	177 152 (17.0%)	0 (0.0%)	0 (0.0%)	104 290 (61.1%)	62 883 (66.3%)	0 (0.0%)	0 (0.0%)	9 978 (61.5%)
Female, total	All ages	1 031 497	452 597	160 677	192 076	115 651	11 109	81 143	18 244
Female	<10	26 820 (2.6%)	13 240 (2.9%)	8821 (5.5%)	0 (0.0%)	0 (0.0%)	327 (2.9%)	4433 (5.5%)	0 (0.0%)
Female	10-14	47 529 (4.6%)	24 593 (5.4%)	14 845 (9.2%)	0 (0.0%)	0 (0.0%)	605 (5.4%)	7486 (9.2%)	0 (0.0%)
Female	15-19	60 318 (5.8%)	34 872 (7.7%)	16 339 (10.2%)	0 (0.0%)	0 (0.0%)	856 (7.7%)	8251 (10.2%)	0 (0.0%)
Female	20-24	67 621 (6.6%)	42 569 (9.4%)	15 852 (9.9%)	132 (0.1%)	1 (0.0%)	1044 (9.4%)	8010 (9.9%)	12 (0.1%)
Female	25-29	72 570 (7.0%)	43 374 (9.6%)	18 052 (11.2%)	843 (0.4%)	36 (0.0%)	1064 (9.6%)	9122 (11.2%)	80 (0.4%)
Female	30-34	73 284 (7.1%)	41 256 (9.1%)	18 076 (11.2%)	3100 (1.6%)	413 (0.4%)	1012 (9.1%)	9134 (11.3%)	293 (1.6%)
Female	35-39	74 558 (7.2%)	42 739 (9.4%)	15 700 (9.8%)	5129 (2.7%)	1525 (1.3%)	1049 (9.4%)	7933 (9.8%)	484 (2.7%)
Female	40-44	74 949 (7.3%)	41 913 (9.3%)	14 142 (8.8%)	7335 (3.8%)	2694 (2.3%)	1028 (9.3%)	7146 (8.8%)	691 (3.8%)
Female	45-49	76 211 (7.4%)	43 223 (9.5%)	11 684 (7.3%)	9117 (4.7%)	4363 (3.8%)	1061 (9.5%)	5904 (7.3%)	859 (4.7%)
Female	50-54	78 497 (7.6%)	44 027 (9.7%)	10 164 (6.3%)	10 865 (5.7%)	6201 (5.4%)	1080 (9.7%)	5135 (6.3%)	1024 (5.6%)
Female	55-59	78 799 (7.6%)	41 505 (9.2%)	9323 (5.8%)	12 547 (6.5%)	8513 (7.4%)	1018 (9.2%)	4711 (5.8%)	1181 (6.5%)
Female	60-64	78 852 (7.6%)	39 286 (8.7%)	7679 (4.8%)	14 287 (7.4%)	11 410 (9.9%)	964 (8.7%)	3879 (4.8%)	1346 (7.4%)
Female	65+	221 488 (21.5%)	0 (0.0%)	0 (0.0%)	128 720 (67.0%)	80 494 (69.6%)	0 (0.0%)	0 (0.0%)	12 274 (67.3%)

Abbreviations: COM, Commercial; FFS, Fee-For-Service; MA, Medicaid Advantage; MCD, Managed Medicaid; Other MCR, Other Medicaid; UNI, Uninsured; VA, Veterans Affairs.

Incident patients were approximately 2.6% of the total patient population, an incidence rate of 0.016%. About 14% of the incident population over the projection period were ≥65 years old (**Figure 2**). Regionally, the Midwest and Northeast exhibited the highest baseline incidence rates at 20 and 18 per 100,000, respectively (**Figure 1**). These rates are 25% to 35% higher than observed incidence rates in the South and the West (15 and 14 per 100,000, respectively). About 47% of incident patients were covered under commercial insurance, followed by Medicare (21%) and Medicaid (20%) (**Table 2**).

**Figure 2.**
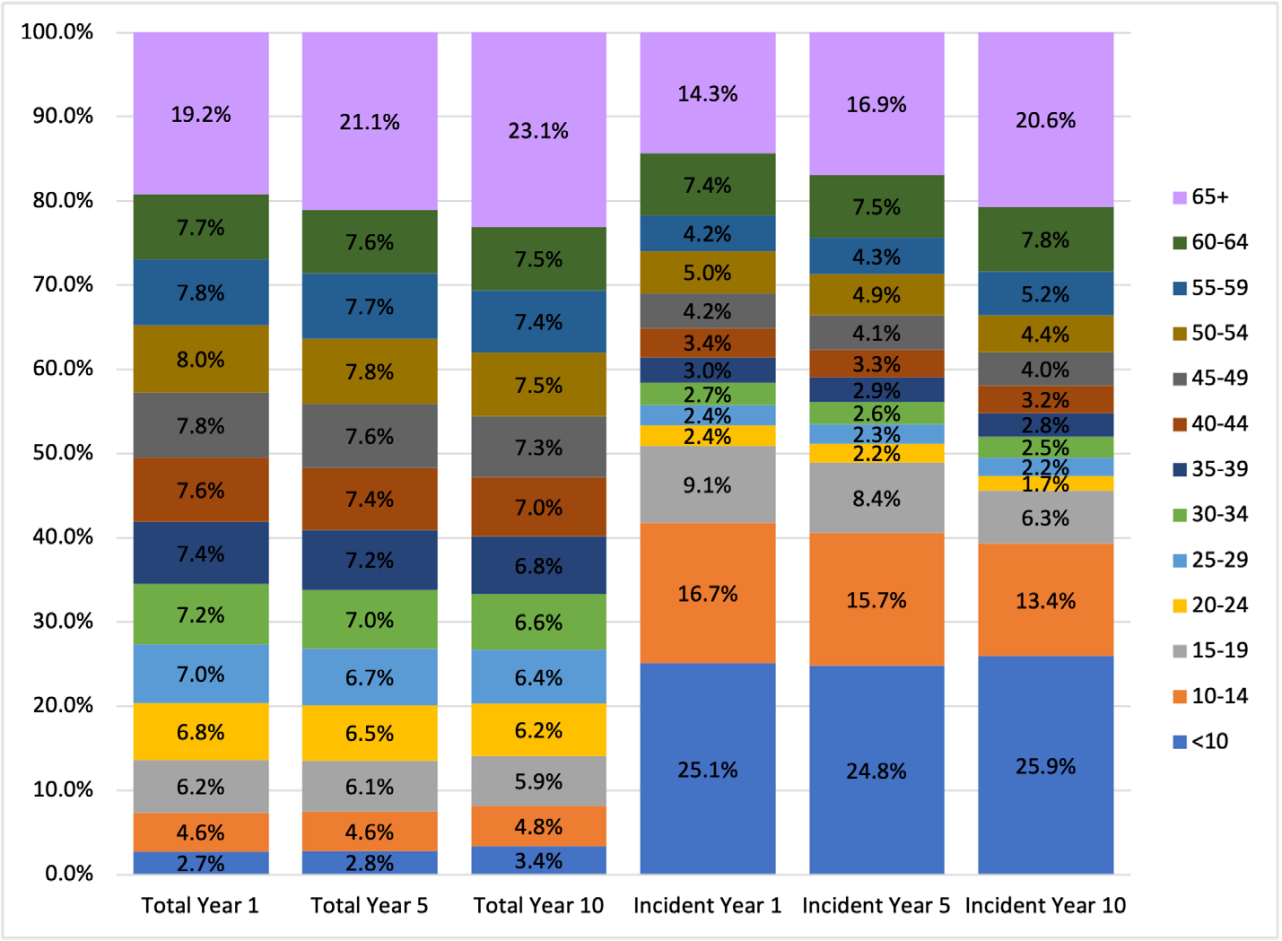
Distribution of Overall and Incident Type 1 Diabetes Populations by Age Band in Model Years 1, 5, and 10

Claims-derived mortality among patients with T1DM were roughly 3 times those of the overall population (2.2%, 2.4%, and 2.6% in Years 1, 5, and 10, respectively, for the T1DM population compared with 0.8%, 0.9%, and 1.0% for the overall population, respectively). However, after incorporating mortality improvement associated with device use, T1DM mortality decreased to around twice that of the overall population (2.0%, 2.0%, and 2.1% in Years 1, 5, and 10, respectively).

### 10-Year Projection

By 2033, we project the US population with T1DM will grow by about 10%, reaching approximately 2.29 million patients from 2.07 in 2024. This growth is attributed to a nearly 50% increase in the number of incident patients over the next decade coupled with improved survival from use of devices. Over the decade, the average age of patients is expected to increase from 47 to 49 years, and the number of patients over age 65 is projected to increase by 33% (**Table 1**). The over-65 growth is primarily influenced by the aging of the US population rather than by increases in T1DM incidence among older Americans.

The largest growth in T1DM incidence is expected in the Northeast. However, due to expected US population growth patterns, T1DM prevalence in the West will increase most (17%).

The racial and ethnic composition of the T1DM population is expected to align with overall US trends. The Hispanic population with T1DM is projected to grow by 18% and the African American population with T1DM by nearly 13%. Although non-Hispanic White patients will continue to constitute the majority of the US T1DM population, that share is projected to decrease from 68% to 66%, and 10-year growth among non-Hispanic White patients with T1DM will be less than 10%, the lowest among all racial and ethnic groups modeled (**Table 1**).

MA and MCD are expected to undergo the most substantial increases in T1DM growth over the next decade, with patient populations growing by 25% and 30% respectively. Projected growth in T1DM among MA-covered patients reflects ongoing, known shifts in Medicare coverage from FFS to MA (**Table 1**).

## DISCUSSION

Our analysis produces somewhat different results than other studies. Recent estimates of T1DM prevalence broken down by age, gender and race/ethnicity were published by Fang et al.^35^ Their work indicated that 4 in every 1000 youths and 5 in every 1000 adults in the US reported having T1DM between 2019 and 2022. Their adult rates are lower compared with our analysis which suggests about 3 T1DM cases per 1000 in youth, 7 cases per 1000 in adults, and 6 cases per 1000 overall. This differential may be explained by the fact that the Fang study was based on self-reported data, which may be susceptible to inaccuracies or low response rates, whereas our study relied on claims data. The Fang study had a response rate of 50% to 60%.

Our estimated T1DM population differed from those studied in other expansive studies of this disease in part due to its national approach. Prior to our study, the most detailed estimates of T1DM prevalence and incidence to date derived from the SEARCH for Diabetes in Youth studies, which examined populations in 10 states between 2001 and 2009.^36^ The SEARCH study observed an increase of 21.1% in the prevalence of T1DM among US youth over that period. However, the SEARCH study did not include data from Northeastern states. By comparison, our analysis projected an increase in the national prevalence of T1DM among patients under 20 years old of 7.6% from 2024 to 2033. It is challenging to compare these 2 analyses’ findings due to the differing time periods and regional distributions studied, but this could suggest that growth in the youth population with T1DM may be slowing over time.

Our nationwide analysis revealed that the Northeast region has the highest T1DM incidence. Regional variation in incidence has been observed in both domestic and international studies. For example, a 2020 study by Mobasseri et al found America had the highest incidence compared with Asia, Africa, and Europe.^37^ Another study among children aged 0 to 4 years of age found Western European regions had the highest incidence compared with other world regions in this age group.^38^ Additionally, a study on US incidence of T1DM from 2001 to 2015 by Rogers et al used more granular census regions and reported increases in incidence were highest in the East South Central region (3.8% per year), followed by the Mountain division (3.1% per year) and then the East North Central region (2.7% per year).^39^ Notably, this study was limited to commercially insured individuals only. By contrast, our broader analysis observed the greatest growth in incidence in the Northeast, at about a 4% increase in incidence annually.

We combined US regional incidence differences with population forecasts that included regional and socioeconomic factors. The results show important differences across regions, payers, and ethnic groups. We found T1DM prevalence tends to vary by income, at least within the Medicare populations, where using patients’ dual eligibility for Medicaid can serve as a proxy for income. This is consistent with several international studies that found more developed countries observe higher incidence and prevalence than less developed countries.^1,37^ It is possible that higher patient income may reduce forgone healthcare services allowing for more complete and accurate coding in clinical or administrative data.

Much existing literature on T1DM prevalence and incidence is based on epidemiological, clinic-based, population-based, prospective birth, case cohort, and cross-sectional studies.^13^ These studies may have limitations such as inadequate representation for the full population, insufficient detail on potential additional contributing factors, limited sample sizes, challenges in control selection, and bias in self-reported data, particularly in survey-based methodologies. By contrast, our study used population-based cross-section data from claims to determine T1DM estimates. Advantages of claims studies include data quality and consistency, clinical validity, ability to link demographic variables, and broad data availability, but we recognize other limitations.^40^ For example, we identified individuals with T1DM using various fields including diagnosis, procedure, and drug codes. These fields may be underreported or misreported. Payment for drugs or devices does not guarantee actual patient use. Indeed, our estimates of device use were based on claims for these devices, but we did not assess adherence, so our estimates may overstate actual, ongoing utilization. Furthermore, our sample of commercial and Medicaid populations were only about 5% and 8% of the respective populations, and, while we believe they are nationally representative, these samples could be biased. In addition, it is possible that the same person could appear more than once if they switched insurers, as there is no common identifier across insurers.

Distinguishing between T1DM and T2DM poses challenges both clinically and epidemiologically. T1DM is the less common condition, and many T1DM cases may be coded as T2DM. Adult-onset T1DM may be especially subject to miscoding due to the incorrect perception that adult cases are rare. According to an article from the American Diabetes Association, misdiagnosis makes up nearly 40% of new cases of T1DM in adults.^41^

CGMs and insulin pumps have become the standard of care under US and other clinical guidelines,^42,43^ and use is increasing.^34^ Their adoption is associated with decreased complications including hypoglycemia, diabetic ketoacidosis, and diabetes-related emergency visits.^34^ Our model incorporated projected increases in uptake for devices over time to reflect their clinical value and recent trends. As device use increases, we anticipate a reduction in complications and, consequently, projected deaths. However, each payer may implement specific coverage criteria or requirements, which can limit or delay access.

Commercially insured patients comprised the largest portion of our modeled population, followed by Medicare and Medicaid. According to the Medicare Local Coverage Determination (LCD) which determines the “reasonable and necessary” criteria for CGM coverage,^44^ CGMs are covered only when the following criteria are met:

1. The beneficiary has diabetes based on ICD-10 codes.
2. The beneficiary is administered insulin 3 or more times daily.
3. The beneficiary’s treatment requires regular adjustment.
4. Within 6 months prior to ordering the CGM, the beneficiary must have an inpatient visit with their treating practitioner confirming that criteria 1, 2, and 3 are met.
5. The beneficiary must have an in-person follow-up every 6 months to assess adherence.

FFS patients who meet the above criteria are eligible for coverage of their devices with few restrictions based on brand or cost. However, access to therapies among commercial insurance plans can differ significantly. Commercial insurance plans may categorize insulin on different formulary tiers, resulting in varying coverage and out-of-pocket costs. Additionally, devices may be subject to insurer approval based on medical necessity criteria.

The mortality loads developed from claims data for patients with T1DM were nearly 3 times those of the general population, consistent with published findings.^45^ T1DM death counts produced by our model were also consistent with previously published mortality studies.^46^ Our modeling explored the expected life-years gained based on mortality improvement from device use over the 10-year projection. We estimate this improvement will result in nearly 360,000 additional life-years, a 2% increase, compared with the baseline scenario. While literature suggests that device use may improve risk factors for comorbidities, there is limited literature available on the quantitative impact that devices may have on mortality.

A recent study published identified a global increase of 60% to 107% in T1DM prevalence from 2021 to 2040.^47^ In contrast, our study suggests a 10% US increase from 2024 to 2033. Our study was limited to the United States, a more developed country. Prevalence of T1DM is higher in more developed countries, but growth in prevalence tends to be higher in less developed countries.^46^ As technology for diagnosing and treating T1DM becomes more widely available, we expect developing countries to show greater T1DM growth.

Our study found lower incidence rates in middle age than in children and young adults, consistent with prior research.^47^ However, viewed as a whole, we observed substantial incidence across middle and older age brackets. As new-onset T1DM is more commonly misdiagnosed as T2DM in adults than in children,^48^ our findings suggest there are public health implications to missing older individuals when considering how best to identify, treat, and support patients with this disease.

Our Year 1 (2024) T1DM population size is based on observed rates from 2019 data. Trending the data to 2024 introduces uncertainties, partly because of the disruption caused by the COVID-19 pandemic. Indeed, all population forecasts involve uncertainty because of economic, demographic, and clinical changes. By example, there have likely been changes in patient outcomes and mortality since 2019 due to the entrance and increased use of closed loop insulin delivery systems which combine CGM and pump.

Race and patients’ dates of death were available only in the FFS data set. The race field is self-reported, introducing potential inaccuracies. Mortality loads for all ages and payers were developed using FFS data, and while these loads are akin to those reported in literature, they may not be appropriate for other coverages. Of course, models are simplifications of reality, and assumptions applied for modeling purposes will likely differ from future experience.

Finally, the commercial databases we used comprise claims primarily from patients covered by large, self-insured employer-sponsored health plans with relatively rich benefits compared with Medicare or Medicaid. These data are recorded for the purpose of payment, not clinical intent, and thus are imperfect when clinical assumptions are applied. Additionally, MA data were available only through 2019, so our initial T1DM identification period was shorter for that market. Finally, we could not access data for certain populations, such as the Medicaid FFS, VA, and Uninsured populations, so we used proxies. Analyses using different years, data sources, methodologies may produce different results.

## CONCLUSION

T1DM impacts 2 million people in the United States. Despite advances in technology and care management, these patients face high comorbidity and mortality risks, and T1DM prevalence continues to grow. But today also sees rapid evolution in our understanding and ability to treat T1DM. Clinicians can now screen for future risk of developing T1DM via blood test; multiple human clinical trials are underway for cell therapies that could end T1DM patients’ reliance on external insulin, and in 2022 the FDA approved the first disease-modifying therapy delaying T1DM onset. Given this rapidly changing landscape, data about the T1DM community is essential to ensure informed decisions by key stakeholders. This study represents a step toward a detailed understanding of the future composition of the T1DM population.

### Disclosures

The authors are employees of Milliman, Inc., an international healthcare consulting and actuarial firm, and Breakthrough T1D, a global type 1 diabetes patient advocacy and research organization.

## Supplementary Material

Online Supplementary Material
